# Efficacy of a new oral chewable tablet containing sarolaner, moxidectin and pyrantel (Simparica Trio™) against induced ascarid infections in dogs

**DOI:** 10.1186/s13071-020-3950-5

**Published:** 2020-03-01

**Authors:** Csilla Becskei, Kristina Kryda, Mirjan Thys, Susan Holzmer, Laurel Bowersock, Tiago Fernandes, Leon Meyer, Craig Reinemeyer, Sean P. Mahabir

**Affiliations:** 1 Zoetis, Veterinary Medicine Research and Development, Mercuriusstraat 20, 1930 Zaventem, Belgium; 2 Zoetis, Veterinary Medicine Research and Development, 333 Portage St., Kalamazoo, MI 49007 USA; 3 Clinvet International (pty) Ltd, Uitsigweg, Bainsvlei, Bloemfontein, 9338 Republic of South Africa; 4 East Tennessee Clinical Research, Inc, 80 Copper Ridge Farm Road, Rockwood, TN 37854 USA

**Keywords:** Roundworms, Immature stages, Adult stages, *Toxocara canis*, *Toxascaris leonina*

## Abstract

**Background:**

Ascarid infections are among the most prevalent intestinal parasitic infections occurring in dogs around the world, with *Toxocara canis* and *Toxascaris leonina* commonly observed. *Toxocara canis* can cause considerable disease in dogs and humans, and year-round prophylactic treatment and control in dogs is recommended. Elimination of immature stages of these parasites before egg-laying will reduce environmental contamination and the risk of infection for both dogs and humans. Studies were conducted to evaluate the efficacy of a novel, oral chewable tablet containing sarolaner, moxidectin and pyrantel (Simparica Trio™) against induced immature adult (L_5_) and adult *T. canis*, and adult *T. leonina* infections in dogs.

**Methods:**

Six negative-controlled, masked, randomized laboratory studies were conducted. Two studies each evaluated efficacy against immature adult (L_5_) *T. canis*, adult *T. canis*, and adult *T. leonina.* Sixteen to 40 dogs were included in each study. Dogs experimentally infected with the target parasite were dosed once on Day 0 with either placebo tablets or Simparica Trio™ tablets to provide minimum dosages of 1.2 mg/kg sarolaner, 24 µg/kg moxidectin and 5.0 mg/kg pyrantel (as pamoate salt). Efficacy was based on the number of worms recovered at necropsy 7–10 days after treatment compared to placebo control.

**Results:**

Based on geometric mean worm counts, efficacy of the sarolaner + moxidectin + pyrantel combination was ≥ 95.2% against immature adult *T. canis*, ≥ 97.3% against adult *T. canis*, and ≥ 89.7% against adult *T. leonina*. There were no treatment-related adverse events in any study.

**Conclusions:**

These studies confirm the efficacy of a single dose of a new oral chewable tablet containing sarolaner, moxidectin and pyrantel (Simparica Trio™) against immature adult and adult *T. canis*, and adult *T. leonina* infections in dogs.

## Background

Ascarid (‘roundworm’) infections are among the most prevalent infections occurring in dogs around the world, with *Toxocara canis* and *Toxascaris leonina* commonly observed. Clinical importance is focused on *T. canis* due to its potential to cause considerable disease in dogs and humans. Dogs can become infected *in utero* with *T. canis* by transplacental larval migration and therefore, infections are common in young puppies [1, 2]. Infections can cause adverse clinical signs, with diarrhea, emesis, abdominal discomfort, enlarged abdomen, and reduced growth rate commonly observed [3]. Infected dogs excrete eggs in their feces, which contaminate the environment and lead to a continued source of infection for both dogs and humans [3]. In human toxocariasis the clinical syndrome observed with *T. canis* infection is dependent on the organs and tissues through which the larvae migrate, and includes visceral, neural and ocular larval migrans, and covert toxocariasis [4].

It is recommended that all puppies be dewormed to remove existing ascarid infections, and that all adult dogs be dewormed at least four times a year. To prevent fecal egg shedding, monthly deworming is recommended, particularly if the zoonotic risk is high, e.g. in dogs sharing homes with young children or immunocompromised individuals [5, 6]. A wide variety of products effective against canine ascarids are available, many of which contain pyrantel as the active ingredient. Pyrantel was first introduced in the 1970’s, and it continues to be used today alone and in combination products. Therefore, the efficacy of pyrantel against canine ascarids is well-established at 5 mg/kg, and more recent publications suggest that its effectiveness has not changed since it was first introduced [7, 8]. Moxidectin, a macrocyclic lactone of the milbemycin group, at a high dosage (2.5 mg/kg) is also approved in a topical spot-on (in combination with imidacloprid) for the treatment of a wide range of gastrointestinal and extra-gastrointestinal nematode infections in dogs [9].

Sarolaner is an isoxazoline ectoparasiticide that provides efficacy against fleas and ticks on dogs for at least one month [10]. Combining moxidectin and pyrantel with sarolaner broadens the efficacy spectrum to include heartworm (*Dirofilaria immitis*) and lungworm (*Angiostrongylus vasorum*) prevention and the treatment and control of intestinal nematodes [7–9]. The studies presented here confirm the efficacy of the novel combination product (Simparica Trio™, Zoetis, Parsippany, NJ, USA) against *T. canis* and *T. leonina* infections in dogs.

## Methods

Six negative-controlled, masked, randomized laboratory studies were conducted. All studies were conducted according to the World Association for the Advancement of Veterinary Parasitology (WAAVP) guidelines for evaluating the efficacy of anthelmintics for dogs and cats [11], the International Co-operation on Harmonisation of Technical Requirements for Registration of Veterinary Medicinal Products (VICH) GL7, “Efficacy of anthelmintics: General requirements” [12], and with VICH GL19 “Efficacy of anthelmintics: Specific recommendations for canines” [13]. Personnel involved in making assessments of efficacy or safety were masked to treatment assignments.

### Animals

Sixteen to 40 dogs were included in each study, depending upon the study design. Dogs were purpose-bred laboratory Beagles or mixed breeds. All dogs had undergone an adequate washout period to ensure that no residual activity remained from any previously administered compounds. Dogs were not allowed to be dewormed within 20 days of inoculation and for any previous deworming, only a short-acting anthelmintic with activity mainly limited to the gastrointestinal tract (e.g. pyrantel) was allowed. The administration of macrocyclic lactones was not allowed.

Dogs were examined by a veterinarian and confirmed to be in good health at the time of enrollment. Dogs ranged in age from 8 to 15 weeks at the time of experimental ascarid inoculation and ranged from 2.7 to 17.2 kg body weight at the time of treatment. Dogs were group housed prior to treatment, and individually housed after treatment. Housing enclosures conformed to accepted animal welfare guidelines [14, 15]. Dogs were fed an appropriate maintenance ration of a commercial canine diet for the duration of the study. Water was available *ad libitum.* Dogs were observed for general health at least once daily throughout the studies.

### Design

Study designs are summarized in Table 1. Two studies each evaluated efficacy against immature adult (L_5_) *T. canis* (Studies 1 and 2), against adult *T. canis* (Studies 3 and 4), and against adult *T. leonina* (Studies 5 and 6). In addition to evaluating efficacy of the combination product, in Study 1 the efficacy of a sarolaner + moxidectin combination was also evaluated with the aim to assess whether these two active ingredients provide sufficient efficacy against immature *T. canis*, or only in combination with pyrantel. In Study, 3 the efficacy of each of the individual active ingredients alone was also evaluated to assess whether there was interference when used in combination. In Study 2, dogs were co-infected with *Ancylostoma caninum* and the methodology and efficacy results are reported in a separate paper [16]. Coinfection was not expected to impact efficacy and that was confirmed by the study results.

**Table 1:** Design of studies conducted to evaluate the efficacy of an oral chewable tablet containing sarolaner, moxidectin and pyrantel pamoate against induced ascarid infections in dogs

Study	Ascarid/Target stage (isolate origin)	Treatment group^a^	*n*	Day of treatment	Day of inoculation^b^	Day(s) of fecal egg counts	Day of worm recovery
1	*Toxocara canis/*immature adults (L_5_) (USA)	Placebo	8	0	− 24	na	7
		SAR + MOX + PYR	8				
		SAR + MOX	8				
2	*Toxocara canis/*immature adults (L_5_) (EU)	Placebo	8	0	− 24	na	7
		SAR + MOX + PYR	8				
3	*Toxocara canis/*adults (USA)	Placebo	8	0	− 49	− 3	7
		SAR + MOX + PYR	8				
		SAR	8				
		MOX	8				
		PYR	8				
4	*Toxocara canis/*adults (EU)	Placebo	8	0	− 57	− 8, − 6, − 4, − 3	7
		SAR + MOX + PYR	8				
5	*Toxascaris leonina*/adults (EU)	Placebo	8	0	− 88	− 8, − 6, − 4, − 3	7
		SAR + MOX + PYR	8				
6	*Toxascaris leonina*/adults (EU)	Placebo	8	0	− 88	− 8, − 6, − 4, − 3	10
		SAR + MOX + PYR	8				

^a^Single oral administration providing minimum dosages of 1.2 mg/kg sarolaner (SAR), 24 µg/kg moxidectin (MOX) and 5 mg/kg pyrantel pamoate (PYR)

^b^Each dog inoculated with L_3_ larvated *T. canis* or *T. leonina* eggs. In Studies 1, 2, 5 and 6 each dog was inoculated with 300 ± 50 eggs, and in Studies 3 and 4 each dog was inoculated with 250–300 (± 50) eggs

*Abbreviations*: USA, United States of America; EU, Europe; na, not applicable

### Experimentally induced ascarid infections

Inoculum size and timing between inoculation of dogs with the infective stage of the target parasite and dosing was set based on the life-cycle of the parasite and established guidelines [13]. In order to ensure that the required minimum number of infected dogs were available at the time of dosing, 4 to 8 additional dogs were inoculated for each study. Ascarids used for experimental inoculation were obtained from naturally infected dogs within approximately 4 years before use in these studies. The isolates were maintained by inoculation of donor dogs at regular intervals.

#### Immature adult (L_5_) *T. canis*

Dogs were inoculated orally with 300 (± 50) infective *T. canis* L_3_ larvated eggs 24 days prior to treatment administration. The *T. canis* strain was cultured from feces obtained from a naturally infected dog in the USA (Study 1) and Italy (Study 2), respectively.

#### Adult *T. canis*

Dogs were inoculated orally with 250–300 (± 50) infective *T. canis* L_3_ larvated eggs 49–57 days prior to treatment administration. In Study 3, the *T. canis* isolate was cultured from the feces of a dog that was artificially infected with larvae collected from a naturally infected dog in the USA. In Study 4, the *T. canis* isolate was cultured from feces obtained from a naturally infected dog in Italy.

#### Adult *T. leonina*

Dogs were inoculated orally with 300 (± 50) infective *T. leonina* L_3_ larvated eggs 88 days prior to treatment administration. The *T. leonina* isolate was collected from naturally infected dogs in Italy (Study 5) and in Germany (Study 6).

### Fecal egg counts and randomization

All studies followed a randomized complete block design. For the pre-patent immature adult L_5_ *T. canis* studies, block was based on pre-treatment body weight, and for the adult *T. canis* and adult *T. leonina* studies block was based on pre-treatment fecal egg counts. Quantitative fecal egg counts were performed using a centrifugation-flotation technique [17].

#### Immature adult (L_5_) *T. canis*

In Study 1, the 24 dogs (12 males and 12 females) with the highest body weight were ranked by body weight within sex and allocated randomly to pen and one of the three treatment groups in each block. In Study 2, the 16 dogs (8 males and 8 females) with the highest body weight were ranked by body weight within sex and allocated randomly to pen and one of the two treatment groups in each block.

#### Adult *T. canis*

In Study 3, the 40 dogs (21 males and 19 females) with the highest pre-treatment (Day-3) *T. canis* fecal egg counts were ranked by fecal egg count and allocated randomly to pen and one of the five treatment groups in each block. In Study 4, the 16 dogs (7 males and 9 females) with the highest pre-treatment (geometric mean of Day-8 to -3) *T. canis* fecal egg counts were allocated randomly to pen and one of the two treatment groups in each block.

#### Adult *T. leonina*

In each study, the 16 dogs (8 males and 8 females in Study 5 and 10 males and 6 females in Study 6) with the highest pre-treatment (geometric mean of Day -8 to -3) *T. leonina* fecal egg counts were allocated randomly to pen and one of the two treatment groups in each block.

### Treatment

In all studies, dogs were dosed on Day 0 with either placebo tablets or Simparica Trio™ tablets, or tablets containing one or two of its three active ingredients (sarolaner, moxidectin or pyrantel pamoate) according to the study design. Each dog received from one to three tablets of the active(s) or the equivalent number of placebo tablets to provide as close as possible to the minimum recommended dosages of 1.2 mg/kg sarolaner, 24 µg/kg moxidectin and 5 mg/kg pyrantel (as pamoate salt) without under-dosing. Body weights obtained within 3 days prior to dosing were used for dose calculation. Placebo and active tablet presentations were similar in order to maintain masking. Food was withheld overnight prior to treatment administration and animals were not fed again until at least 4 hours after treatment. All doses were administered by hand pilling to ensure accurate dosing. Each dog was observed for several minutes after dosing for evidence that the dose was swallowed.

### Necropsy and worm recovery

Food was withheld for approximately 15 hours prior to euthanasia. Dogs were humanely euthanized by intravenous injection of an approved euthanasia solution containing phenobarbital sodium at the label dosage. After euthanasia the entire gastrointestinal tract from distal esophagus to rectum was removed, split longitudinally, and the mucosal surface scraped twice to remove attached ascarids. Additional details regarding the washing and sieving of the gastrointestinal contents and the material collected from scraping, as well as any additional tissue processing for worm recovery, are described below for each study. The contents of the sieves were rinsed, preserved in formalin and examined under magnification to identify and count recovered worms.

#### Immature adult (L_5_) *T. canis*

In both studies, the gastrointestinal contents and the scrapings were washed over a sieve with a maximum aperture size of 150 µm. The scraped stomach and small intestine were then soaked in 0.9% saline solution and incubated at approximately 90–100 °F (32.0–38.2 °C) for approximately 2 to 4 hours. After incubation, the soaked tissue was stripped twice to remove any released ascarids and the stripped material was washed over the sieve.

#### Adult *T. canis*

In Study 3, the gastrointestinal contents and scrapings were washed over a sieve with an aperture size of 150 µm, and in Study 4 the contents and scrapings from the stomach and small intestine were washed over a sieve with an aperture size of 150 µm, whereas the contents and scrapings from the large intestine were washed over a sieve with an aperture size of 300 µm.

#### Adult *T. leonina*

In both studies, the contents and scrapings from the stomach and small intestine were washed over a sieve with an aperture size of 150 µm, whereas the contents and scrapings from the large intestine were washed over a sieve with an aperture size of 300 µm.

### Statistical analysis

The experimental unit was the individual dog. Percent efficacy relative to placebo was calculated using geometric means (back-transformed least square means) based on the formula [(C − T)/C] × 100, where C is the mean worm count for the placebo group and T is the mean worm count for the treated group.

Worm counts were transformed by the log_e_ (count + 1) transformation prior to analysis in order to stabilize the variance and normalize the data. Transformed counts were analyzed using a general mixed linear model (SAS 9.4, Cary NC) that included the fixed effect of treatment, and the random effects of block and error, except for Study 3, which included the random effects of room, block within room, and error (because dogs were housed in separate rooms). Testing was two-sided at the significance level α = 0.05.

## Results and discussion

There were no mortalities and no treatment-related adverse reactions in any study. The most frequently observed abnormal health events in all treatment groups in all studies were gastrointestinal signs commonly associated with intestinal parasitism (i.e. emesis, diarrhea, blood and/or mucus in the feces, and/or ascarids in the feces).

Infection was adequate in all studies with 5 or more worms found in at least 6 placebo-treated dogs in each study. Efficacy results are summarized in Table 2.

**Table 2:** Efficacy of a single dose of an oral chewable tablet containing sarolaner, moxidectin and pyrantel pamoate against induced ascarid infections in dogs

Study	Ascarid/Target stage (isolate origin)	Treatment group^a^	*n*	Geometric mean worm count	Efficacy compared to placebo	
					% Efficacy	Test statistic
1	*Toxocara canis/*immature adults (L_5_) (USA)	Placebo	8	33.3	–	–
		SAR + MOX + PYR	8	1.6	95.2	*t*_(14)_ = 6.32 *P < *0.0001
		SAR + MOX	8	8.4	74.7	*t*_(14)_ = 3.16 *P* = 0.0070
2	*Toxocara canis/*immature adults (L_5_) (EU)	Placebo	8	15.2	–	–
		SAR + MOX + PYR	8	0.3	97.9	*t*_(14)_ = 5.61 *P* = 0.0008
3	*Toxocara canis/*adults (USA)	Placebo	8	11.5	–	–
		SAR + MOX + PYR	8	0.1	99.2	*t*_(26)_ = 7.99 *P < *0.0001
		SAR	8	7.3	36.7	*t*_(26)_ = 1.35 *P* = 0.1869
		MOX	8	0.8	92.8	*t*_(26)_ = 6.29 *P < *0.0001
		PYR	8	0	100	*t*_(26)_ = 8.27 *P < *0.0001
4	*Toxocara canis/*adults (EU)	Placebo	8	24.4	–	–
		SAR + MOX + PYR	8	0.6	97.3	*t*_(7)_ = 10.40 *P* = 0.0001
5	*Toxascaris leonina*/adults (EU)	Placebo	8	25.0	–	–
		SAR + MOX + PYR	8	2.6	89.7	*t*_(7)_ = 7.98 *P < *0.0001
6	*Toxascaris leonina*/adults (EU)	Placebo	8	20.1	–	–
		SAR + MOX + PYR	8	0.1	99.6	*t*_(7)_ = 17. 02 *P < *0.0001

^a^Single oral administration providing minimum dosages of 1.2 mg/kg sarolaner (SAR), 24 µg/kg moxidectin (MOX) and 5 mg/kg pyrantel pamoate (PYR)

### Immature adult (L_5_) *T. canis*

In Study 1, the geometric mean worm count for the placebo group was 33.3, and all 8 placebo-treated dogs had 11 or more worms. Reduction in geometric mean worm counts for the sarolaner + moxidectin + pyrantel combination product group was 95.2% (*t*_*(*14)_ = 6.32, *P* *<* 0.0001), and for the sarolaner + moxidectin group 74.7% (*t*_(14)_ = 3.16, *P* = 0.0070) when compared to placebo. Geometric mean worm count for the sarolaner + moxidectin + pyrantel combination product group was significantly lower than for the sarolaner + moxidectin group (*t*_(14)_ = − 3.16, *P* = 0.0069). In Study 2, the geometric mean worm count for the placebo group was 15.2, and 7 of the 8 placebo-treated dogs had 5 or more worms. Reduction in geometric mean worm counts for the sarolaner + moxidectin + pyrantel group was 97.9% (*t*_(7)_ = 5.61, *P* = 0.0008).

The results of these studies confirm the efficacy of sarolaner + moxidectin + pyrantel against immature adult *T. canis* in dogs. Study 1 provided evidence that for sufficient efficacy (> 90%) against immature adult *T. canis,* pyrantel is essential in the combination, as the efficacy of moxidectin and sarolaner alone was only 74.7%. Effective treatment of immature stages is becoming more important, as an ELISA for coproantigen detection has recently been developed [18, 19]. This test has the potential to diagnose *T. canis* infection in the pre-patent period when standard fecal microscopic examinations are unable to detect the infection due to the absence of egg shedding. A product with confirmed efficacy against immature adult *T. canis* is of critical importance to control this important zoonotic parasite. *Toxocara canis* eggs can remain infective for many years in the environment thus leading to a continued source of infection for both dogs and humans [20]. Therefore, elimination of worms before they can mature and begin egg-laying will reduce environmental contamination and should decrease the risk of both human and animal infection.

### Adult *T. canis*

In Study 3, the geometric mean worm count for the placebo group was 11.5, and 6 of the 8 placebo-treated dogs had 9 or more worms. Compared to placebo, the reduction in geometric mean worm counts for the combination product group was 99.2% (*t*_(26)_ = 7.99, *P < *0.0001). The reduction in geometric mean worm counts for the individual actives were: 36.7% (*t*_(26)_ = 1.35, *P* = 0.1899) for sarolaner alone, 92.8% (*t*_(26)_ = 6.29, *P < *0.0001) for moxidectin alone, and 100% (*t*_(26)_ = 8.27, *P < *0.0001) for pyrantel alone when compared to placebo. The geometric mean worm counts for sarolaner alone were significantly different compared to moxidectin and pyrantel alone (1.51 ≤ *t*_(26)_ ≤ 2.12, *P < *0.0001), but not for moxidectin compared to pyrantel alone (*t*_(26)_ = 1.98, *P* = 0.0587). In Study 4, the geometric mean worm count for the placebo group was 24.4, and all 8 placebo-treated dogs had 5 or more worms. Reduction in geometric mean worms counts for the combination product group was 97.3% (*t*_(7)_ = 10.40, *P* = 0.0001).

The results of these studies confirm the efficacy of the combination product against adult *T. canis* in dogs. In Study 3, the 99.2% efficacy provided by the combination was not significantly different (*t*_(26)_ = 0.28, *P* = 0.7791) than the 100% efficacy provided by pyrantel alone, confirming that there was no interference on the efficacy of pyrantel when administered in combination with sarolaner and moxidectin. In the same study, a single oral dose of moxidectin alone at 24 µg/kg provided 92.8% efficacy against adult *T. canis.* To the authors’ knowledge this is the first study that evaluated the efficacy of orally administered moxidectin against adult *T. canis.* Previous published studies have shown that moxidectin was not effective against adult *T. canis* (0.0%) when administered once subcutaneously at a dosage of 0.17 mg/kg [21], and was effective (≥ 94.0%) when administered once topically at a dosage of 2.5 mg/kg [9].

### Adult *T. leonina*

In Study 5, the geometric mean worm count for the placebo group was 25.0, and all 8 placebo-treated dogs had 15 or more worms. Reduction in geometric mean worm counts for the combination product group was 89.7% (*t*_(7)_ = 7.98, *P < *0.0001). In Study 6, the geometric mean total worm count for the placebo group was 20.1, and all 8 placebo-treated dogs had 7 or more worms. Reduction in geometric mean worm counts for the combination product group was 99.6% (*t*_(7)_ = 17.02, *P < *0.0001).

The results of these studies confirm the efficacy of the combination product against adult *T. leonina* in dogs.

### Clinical benefits of the combination product

Ascarids, hookworms, heartworms, fleas and ticks are parasites that commonly infect dogs. Due to their potential to cause significant clinical disease, year-round treatment and prevention of these parasites is recommended [5, 6, 22–27].

The studies presented here confirm that pyrantel in combination with sarolaner and moxidectin is effective against immature adult (L_5_) and adult *T. canis*, and adult *T. leonina* in dogs. The efficacy of Simparica Trio™ was similarly high in all studies, including against the two different isolates of each species used that were collected from geographically distinct regions (i.e. USA and Europe), confirming similar susceptibility of the isolates against the active ingredients. The dosage of pyrantel in Simparica Trio™ was based on the well-established and widely used dose rate of 5.0 mg/kg [28]. The 100% efficacy of pyrantel alone demonstrated against adult *T. canis* in Study 3 is consistent with more recent publications [8] showing that its efficacy against canine ascarids has not changed since it was first introduced. This study also demonstrated that a single oral dose of moxidectin alone at 24 µg/kg is effective against adult *T. canis* in dogs. As Study 1 demonstrated, this dosage of moxidectin in combination with sarolaner is however suboptimal against immature adults of *T. canis*. To provide reliable efficacy against this stage, pyrantel is needed in the combination. Moxidectin at a single oral dose of 3 µg/kg was shown to be effective against susceptible *Dirofilaria immitis* isolates [29], and more recent studies have confirmed its efficacy in the prevention of canine heartworm (L_4_ stages) and lungworm (L_5_ stage) disease at 24 µg/kg in combination with pyrantel and sarolaner [30, 31]. The dosage of moxidectin in Simparica Trio™ has been selected in dose titration studies against these vascular nematodes [31, 32]. Efficacy of sarolaner against fleas and ticks when administered in combination with moxidectin and pyrantel has also been recently demonstrated [33–35]. The dosage of sarolaner in Simparica Trio™ has been selected based on dose titration and confirmation studies against these ectoparasites [35].

An orally administered chewable tablet that combines sarolaner, moxidectin, and pyrantel should provide a convenient method for the pet owners to treat and control some of the most common internal and external parasites infecting dogs.

## Conclusions

These studies confirm the efficacy of a single oral dose of a new chewable tablet containing sarolaner, moxidectin and pyrantel pamoate (Simparica Trio™) against immature adult (L_5_) and adult *T. canis*, and adult *T. leonina* infections in dogs.

## Data Availability

Data upon which the conclusions are based are provided within the article.

## Abbreviations

EU: Europe
na: Not applicable
MOX: Moxidectin
PYR: Pyrantel pamoate
SAR: Sarolaner
